# Vegetable and Fruit Consumption and Psychological Distress: Findings from Australian National Health Survey Data, 2011–2018

**DOI:** 10.3390/ijerph22071037

**Published:** 2025-06-28

**Authors:** Kerri M. Gillespie, Melanie J. White, Eva Kemps, Selena E. Bartlett

**Affiliations:** 1 School of Clinical Sciences, Faculty of Health, Queensland University of Technology, Kelvin Grove, QLD 4059, Australia; selena.bartlett@qut.edu.au; 2 School of Psychology and Counselling, Faculty of Health, Queensland University of Technology, Kelvin Grove, QLD 4059, Australia; melanie.white@qut.edu.au; 3 College of Education, Psychology and Social Work, Flinders University, Bedford Park, SA 5042, Australia; eva.kemps@flinders.edu.au

**Keywords:** diet, vegetables, psychological distress, chronic illness

## Abstract

This study aims to determine the association between vegetable and fruit consumption and other lifestyle factors and the prevalence of psychological distress. Sex differences in these relationships are also examined. Data from 45,717 participants aged 18 and older, obtained via the Australian Bureau of Statistics National Health Survey (years 2011–12, 2014–15, and 2017–18), were analysed using logistic regression with jackknife parameter estimation. Vegetable consumption was inversely related to psychological distress. Eating two servings of fruit per day was associated with lower distress, but additional servings did not have the same effect. When stratified by sex, only women benefited from fruit consumption. When accounting for long-term health conditions, the sex difference in distress was ameliorated. Older age, higher exercise levels, and not smoking were significantly associated with lower distress. Frequency of alcohol consumption was inversely associated with distress. Lower-income groups consumed greater quantities of fruits and vegetables than higher-income individuals. Vegetable consumption appears to be more strongly associated with mental health than fruit consumption. Chronic disease symptom management may be one way of addressing sex differences with regard to distress levels. The differential impact of dietary components on men and women requires further investigation to determine if the effects are due to a true biological difference or unidentified confounders.

## 1. Introduction

Mental health disorders are a serious and growing health concern worldwide, accounting for an estimated 16% of global disease burden [1]. Psychological distress, characterised by symptoms of depression, anxiety, and stress, is frequently used as an indicator of a population’s mental health. Distress has been linked to numerous adverse outcomes, including increased risk of suicide [2], chronic disease [3], all-cause mortality [4], and poor academic performance [5]. To assist in the prevention, identification, and treatment of mental illness, it is imperative to understand associated contributing and protective factors.

It is now well understood that diet and other modifiable lifestyle factors have a significant influence on our mental health. Dietary composition and its impact on physical and psychiatric wellbeing has been a focus of health research for decades [6]. The Western diet, defined by high-fat, high-sugar, and ultra-processed foods, has been associated with metabolic disease, cardiovascular disease, increased cancer risk, dementia, and mental illness [6,7,8]. Ultra-processed foods (UPFs), created through industrial processes designed to extend product shelf-life, are often high in sugars, fats, emulsifiers, and preservatives [9,10]. UPFs have been linked to mental illness, chronic disease, and all-cause mortality [9,11]. Excess free and added sugars have also been linked to systemic inflammation, obesity, chronic disease risk, and increased depression [12,13,14,15,16]. Conversely, Mediterranean and Nordic diets (which include greater quantities of minimally processed plant foods, fish, legumes, and nuts, and lower amounts of added sugars, red meat, and dairy products) are frequently associated with lower rates of cardiovascular disease, diabetes, and dementia [17,18,19,20].

Vegetable and fruit intake has repeatedly shown an inverse relationship with depression, anxiety, and distress [21]. There is also evidence that increased fruit and vegetable consumption may be protective against the detrimental impacts of UPFs and excess sugar consumption [22]. However, when investigating fruits and vegetables separately, conflicting findings have been observed [23]. Some research has suggested a stronger positive association between psychological wellbeing and vegetables compared to fruit [24]. Other research has identified a significant negative correlation between depressive symptoms and fruit consumption, but not vegetable consumption [25]. A large population study in Spain found an inverse dose–response relationship between depression and fruit and nut consumption, but found no association between depression and vegetable intake [26].

Nguyen and colleagues [27] found that, in fully adjusted models, moderate consumption was associated with reduced distress, but this relationship was no longer significant at higher levels of intake. Sex differences have also been observed, with healthy diets inversely related to depressive symptoms in women, but not in men [28]. When men and women were analysed separately, Nguyen and colleagues [27] also found an association between fruit and vegetable consumption and incidence of psychological distress in women only.

The disparities observed in these papers may be due to the statistical techniques used and the choice of covariates included in the analyses. Physical inactivity, increased smoking, and increased alcohol intake are other modifiable lifestyle factors that have also been associated with poor mental health [29]. A number of studies found that relationships between fruit and/or vegetables and mental health were slightly or completely attenuated when controlling for lifestyle, demographic, health, and anthropometric variables such as physical activity, BMI, depression history, socioeconomic factors, chronic illness, and smoking status [30,31,32]. Physical activity in particular has frequently been associated with physical and mental health. An umbrella review of systematic reviews found that all forms of physical activity intervention were beneficial for depression and anxiety [33]. A longitudinal study of adults in Hawaii found that physical activity was positively related to mental health, regardless of fruit and vegetable consumption [34].

The relationships between modifiable lifestyle variables, and how these differentially impact mental health, remain poorly understood. The differential impacts of fruits and vegetables, and the influence of sex on these relationships, are also unclear [35]. Individuals who eat a healthier diet tend to also practice other healthier lifestyle behaviours [36], making it challenging to determine the independent effect of diet on mental health. We therefore need to investigate multiple lifestyle factors and their association patterns to better understand the impacts of individual behaviours on mental health. The current study aims to examine the associations between vegetable and fruit consumption and psychological distress using three Australian population datasets, while also adjusting for other modifiable lifestyle and sociodemographic variables. We also explore the sex differences in these relationships and changes in relationships over time.

## 2. Materials and Methods

### 2.1. Study Design

The data used in this study were obtained from three National Health Surveys (2011–2012; 2014–2015; and 2017–2018) collected by the Australian Bureau of Statistics (ABS). Each survey contained data from its own cohort sample of randomly selected households in Australia. Data from 45,717 adults aged 18 and over were included in the analysis. These years were chosen for optimal comparability. Earlier iterations of the NHS were excluded because they did not include variables considered vital to the analysis, such as income. Earlier versions of the survey did not include replicate variables to enable weighting.

### 2.2. Primary Objectives

1. To investigate the association between vegetable and fruit consumption and the prevalence of psychological distress;
2. To investigate the association between other modifiable lifestyle factors and sociodemographic variables and the prevalence of psychological distress;
3. To evaluate sex differences in the relationships between lifestyle factors and psychological distress.

### 2.3. Assessment of Psychological Distress

The NHS measured psychological distress using the Kessler Psychological Distress Scale (K10). The K10 is a widely used and validated 10-item questionnaire designed to produce a global measure of distress based on the severity of anxiety and depression symptoms experienced in the preceding 30 days [37,38]. Questions included “about how often did you feel tired out for no good reason”; “about how often did you feel hopeless”; and “about how often did you feel so restless you could not sit still?” These questions were answered on a five-point scale of 1 (none of the time) to 5 (all of the time).

The current study used the cutoffs recommended by the ABS: 10–15 (low distress); 16–21 (moderate distress); 22–29 (high distress); and 30–50 (very high distress). Previous research has found that a cutoff of 20 or more showed good sensitivity (0.66 to 0.80) and specificity (0.081 to 0.92) for a depressive and/or anxiety disorder [39,40]. Other research identified 24 as an optimal cutoff [41]. We therefore used the midway cutoff of 22 (incorporating high and very high levels of distress) to form a two-category outcome variable of psychological distress for the purposes of binary logistic regression.

### 2.4. Independent Variables

The primary independent variables were vegetable consumption and fruit consumption, both measured as the usual number of servings consumed per day. The 2013 National Health and Medical Research Council (NHMRC) dietary guidelines were used when analysing fruit and vegetable consumption [42]. The NHMRC recommendations for daily vegetable consumption are as follows: 5 servings for women aged 18 and older; 5.5 servings for men aged 18, and men aged 51 to 70; 6 servings for men aged 19 to 50; and 5 servings for men aged 71 and older. Recommendations for daily fruit consumption are 2 servings per day for all adults. Fruit and vegetable variables were condensed into categories based on NHMRC recommendations, and population sizes within each category: 1 serving or less; 2 servings; 3 to 4 servings; 5 or more servings. Other variables included in the analyses were age, sex, year (of survey), level of exercise (undertaken in the last week), frequency of alcohol consumption, whether the respondent exceeded alcohol consumption guidelines (yes or no), smoking status, income (quintile), highest level of educational attainment, bodily pain (experienced in the last four weeks), number of current long-term conditions, self-assessed health, and body mass index (BMI). Participants who drank more than 10 drinks per week were labelled as having exceeded alcohol guidelines, as per the NHMRC revised guidelines [43]. Self-assessed health was not included in the final model, as it may have been significantly influenced by psychological distress, and may have been too closely related to bodily pain and chronic conditions to be included in the same model. Marital status was not available for each year and so was excluded from the model.

### 2.5. Data Analysis

Data was weighted using jackknife parameter estimation using STATA version 17.0 (StataCorp LLC, College Station, TX, USA). The ABS provided 60 replicate weights and a person weight to adjust for sampling methods and complex survey design characteristics. Missing responses were imputed by the ABS using “hot decking” imputation [44]. This is where a missing response is replaced by the response of another participant, chosen based on matching characteristics such as age, sex, and BMI. In preliminary analysis, chi-square tests were employed to investigate the relationships between all variables. Independent variables that were significantly related to psychological distress were included in the final model. Multivariable logistic regression modelling was conducted to determine the associations between lifestyle and sociodemographic variables and psychological distress using odds ratios. Model 1 (minimally adjusted) adjusted for age, sex, and year. Model 2 (lifestyle adjusted) adjusted for age, sex, year, exercise level, smoking status, and alcohol consumption. Model 3 (fully adjusted) adjusted for age, sex, year, exercise level, smoking status, alcohol consumption, income, education, bodily pain, and number of long-term current conditions.

## 3. Results

### 3.1. Demographic Characteristics

Table 1 presents the characteristics of the study population. Of the 45,717 people included, 12.6% of participants (*n* = 5764) had high or very high levels of psychological distress. Just over 50% consumed the recommended daily servings of fruit, and 21.1% consumed more. Only 7.7% of participants consumed the recommended daily servings of vegetables; between five and six servings per day depending on age [42], although closer to 10% consumed at least five servings. The proportion of people adhering to vegetable and fruit guidelines, and prevalence of psychological distress, all increased over the time periods captured by the three NHS surveys. These data were collected before the COVID-19 pandemic and are therefore not influenced by its impact on mental health.

### 3.2. Correlates of Psychological Distress

Initial bivariate analyses showed that all lifestyle factors were significantly associated with psychological distress (see Table 2). BMI did not add to the model, and so was excluded from the final analysis. Frequency of alcohol consumption was more strongly associated with psychological distress and was therefore chosen over alcohol consumption (exceeding recommended guidelines) to be included in the final model.

Servings of vegetables consumed remained significantly associated with psychological distress in the final model. Those consuming one or fewer servings of vegetables per day had 1.3 times the odds of distress compared to those consuming two servings, 1.5 times the odds of those consuming three to four servings, and 1.6 times the odds of distress compared to those consuming five or more servings per day. Consuming one or fewer servings of fruit led to a small increase in odds of distress (1.1 times) compared to that observed in respondents who consumed two servings. Higher consumption of fruit showed no significant difference from one or fewer servings in terms of psychological distress.

Table 3 outlines the results of the multivariable logistic regression models. The inverse relationship between age and distress became stronger with increasing adjustment. The odds of distress reduced markedly with age, with those aged 18 to 29 having more than nine times greater odds of psychological distress compared to individuals aged 75 and over. Exercise was also seen to reduce the odds of distress, although there was little difference observed between moderate and high levels of exercise. Frequency of alcohol consumption was inversely associated with psychological distress. Those who were current or ex-smokers had higher odds of distress than those who had never smoked.

### 3.3. Stratefied Analysis by Sex

Table 2 highlights the significant association of sex with all variables other than self-assessed health. Women were more likely to be sedentary, overweight, have higher educational attainment, suffer from long-term health conditions, and experience bodily pain, whereas men were more likely to drink regularly and smoke, and were disproportionately represented in the highest 40% of incomes. Women experienced higher rates of distress than men in the minimally adjusted or lifestyle-adjusted models (Table 3). However, this difference was not observed in the fully adjusted model. When regression models were stratified by sex (Table 4), the association between vegetable consumption and psychological distress was stronger in women. In the fully adjusted model, the significant association between fruit consumption and psychological distress was only observed in women. Conversely, the significant relationship between frequency of alcohol consumption and distress was only observed in men.

## 4. Discussion

Our findings replicate and extend upon existing literature relating to the impact of diet and other lifestyle factors on psychological distress. In a large, population-level study of Australian adults, adhering to the NHMRC guidelines for vegetables and fruit consumption was significantly associated with reduced psychological distress. Vegetable consumption was more strongly associated with reduced distress than fruit consumption. Consuming even small amounts of vegetables (two to four servings per day) was associated with lower odds of distress. Psychological distress was also associated with a sedentary lifestyle, younger age, smoking, infrequent alcohol consumption, low SES, bodily pain, and the presence of long-term health conditions.

Our findings resembled those of Nguyen and colleagues [30] who found a relationship between fruit and vegetable consumption, but only at low levels. We found the following pattern in fruit consumption for women: those consuming more than the recommended two servings had distress comparable to that of those consuming one or no servings of fruit. For men, fruit consumption had no beneficial impact on distress levels, regardless of the quantity of fruit consumed. A similar relationship was seen for vegetables, but only in men who consumed five or more servings. Women saw a significant and linear inverse relationship between vegetable consumption and distress. Prior studies have reported similar findings that depression and distress in females was more greatly influenced by fruit consumption than in males [45,46]. This could potentially reflect sex-specific differences in the impact of fructose on physiology and behaviour, which has been frequently observed in rat models [47,48]. Women also started from a much higher initial distress level.

Nguyen and colleagues [30] posited that their U-shaped relationship may be due to those consuming larger quantities of fruits and vegetables having a higher socioeconomic status and more favourable health behaviours. We did not find an inverse relationship between fruit or vegetable consumption and SES. The bottom 40% of income earners were seen to consume three or more servings of fruits and vegetables at a higher rate than the top 40% of income earners. Supplementary Table S1 also shows that a higher proportion of those in the bottom 40% for income also had higher rates of adherence to both fruit and vegetable guidelines. This is inconsistent with much existing research [49], including findings from the 1995 NHS survey [50].

Unexpectedly, there was an association between increased frequency of alcohol consumption and lower levels of distress. When alcohol consumption (exceeding guidelines, yes/no) was included instead of frequency, findings were similar, with drinking more than recommended guidelines associated with a small reduction in distress. This was dissimilar to previous studies, which found psychological distress to be associated with higher alcohol consumption [51,52]. When stratified by sex, this inverse relationship was not observed in women. Men were more likely to drink more frequently, to exceed the recommended drinking guidelines, and had lower rates of psychological distress, which may have favoured a relationship between drinking and low distress. Alcohol consumption is also associated with higher incomes, which would also favour lower distress. Higher incomes for men may also explain the higher male drinking rates. We did not investigate alcohol consumption in terms of the severity or regularity of very high consumption, meaning that frequent binge drinkers were incorporated into the same category as those who may drink no more than one standard drink in a sitting, or may exceed drinking guidelines less frequently. There is some evidence that frequent binge drinking may have a more detrimental impact on mental health [53] than lower levels of drinking that still exceed the recommended guidelines.

Similarly to previous studies [52,54], distress was seen to decrease with age. This effect was strengthened when controlling for other lifestyle factors and became even more prominent when controlling for sociodemographic factors. Consumption of fruit and vegetables was also seen to increase with age, with the highest consumption reported amongst individuals aged 60 and above.

When controlling for all variables, low and sedentary lifestyles increased the odds of distress by 1.31 and 1.61, respectively. There did not appear to be a significant difference between moderate and high levels of exercise in terms of distress, indicating that even moderate activity can be beneficial for mental health. Being a smoker was associated with 2.07 times higher odds of distress, and an ex-smoker had 1.2 times the odds of distress compared to someone who had never smoked. This may be because people with mental health issues tend to smoke at higher rates. However, longitudinal studies investigating the impact of smoking or smoke exposure on incidence of mental illness suggest a causal relationship [32]. The largest effect on distress was the presence of long-term health conditions. Having one or two conditions nearly doubled the odds of distress. Having six or more increased the odds of distress by over 12 times.

A number of previous studies have identified a significant sex difference, with women experiencing greater levels of anxiety, depression, and psychological distress [52,55,56]. Our study similarly uncovered higher levels of distress in women in the minimally adjusted or lifestyle-adjusted models. However, when including both SES (income and education) and health factors (long-term conditions and bodily pain) in the model, we no longer observed significantly higher levels of distress in women. Overall, women earned less money than men, which could lead to higher distress in women. However, when removing health and SES factors one by one, only the removal of long-term conditions saw women again having significantly higher odds (1.2) of distress. Women in our estimated population were much more likely to experience three or more long-term health conditions, and were less likely to have no long-term health conditions compared to men. This over-representation was observed in each survey year included in the dataset. The findings are also similar to the results of the 2021 Australian census, which found that women were more likely to suffer from arthritis, asthma, cancer, lung disorders, and dementia [57]. These increased rates of illness may be related to women living longer and therefore being more likely to develop these diseases before death. To explain the sex disparity in mental illness, it may be beneficial to further investigate women’s experiences of chronic disease, access to care, and health-seeking behaviours.

## 5. Strengths and Limitations

The authors carefully considered all potential covariates and included a significant number that have potentially confounding effects on mental health. This provided a more reliable indicator of the impact of diet on psychological distress. We also investigated fruit and vegetable consumption separately, and utilised an ordinal variable with four levels to enable investigation of the pattern of these relationships. However, the study has several limitations. Incorporating three different datasets meant that we had to exclude or alter some variables for compatibility. In some instances, this required the collapsing of variables into fewer levels. Another major limitation of the study was the lack of dietary information other than details of respondents’ fruit and vegetable consumption. Incorporating other dietary components such as individual macronutrients, total energy intake, junk food, or sugar-sweetened beverage consumption would have enabled us to control for the negative impacts of high-fat/high-sugar diets. Data was collected through self-report, which increases the risk of recall bias, social desirability bias, participant misclassification of fruits or vegetables, and different interpretations of portion sizes.

Additionally, different fruits and vegetables contain various compositions of vitamins, minerals, fibre, and phytochemicals [58]. Further investigation of different forms of fruit and vegetable intake could identify the types of fruits and vegetables optimal for reducing psychological distress and improving health and wellbeing. Other potential confounders that would have been beneficial to investigate were also not included in the datasets. The most noteworthy of these was sleep, which has been identified as a significant risk factor for mental illness [32]. Adverse childhood experiences have also been strongly linked to adult mental illness [59]. Data were collected before the onset of the COVID-19 pandemic, which likely significantly increased rates of psychological distress worldwide. Incorporating data collected during this period could have introduced additional confounding. This would have been difficult to control for, as the impacts of COVID-19 on psychological distress may have differed markedly based on location, home environment, vocation, and pre-existing physical and mental health conditions. Variables such as ethnicity, history of mental illness, medications prescribed for mental illness, and frequency of bodily pain would have been useful to include. Findings across this seven-year period are therefore more likely to be robust and comparable, and less likely to have been influenced by significant environmental factors.

## 6. Conclusions

The study found that the consumption of two or more servings of vegetables a day may reduce the prevalence of psychological distress in adults. Consuming the recommended two servings of fruit per day was associated with improved mental health for women, but the benefits of fruit consumption for men is not clear and needs to be further examined. Quitting smoking and partaking in at least moderate- or high-intensity, regular exercise is also important for mental health. The strong association between long-term health conditions, sex, and psychological distress may, at least partially, explain the sex differences in the prevalence of distress. Treatment of chronic disease symptoms should be looked at as a way of potentially reducing distress and minimising the sex inequity observed in rates of psychological distress.

## Figures and Tables

**Table 1:** Demographic characteristics of 45,717 respondents—unweighted.

Item		Number (%) of Respondents
Psychological distress		
	Low distress	30,862 (67.5%)
	Moderate distress	9094 (19.9%)
	High distress	3832 (8.4%)
	Very high distress	1914 (4.2%)
Usual daily servings of vegetables		
	1 or less	12,854 (28.1%)
	2 servings	12,714 (27.8%)
	3 to 4 servings	15,725 (34.4%)
	5 or more	4424 (9.7%)
Usual daily servings of fruit		
	1 or less	22,690 (49.6%)
	2 servings	13,323 (29.1%)
	3 to 4 servings	8656 (18.9%)
	5 or more	1048 (2.3%)
Year		
	2012	15,387 (33.7%)
	2015	14,469 (31.6%)
	2018	15,861 (34.7%)
Sex		
	Male	21,068 (46.1%)
	Female	24,649 (53.9%)
Age		
	18–29	6975 (15.3%)
	30–44	12,617 (27.6%)
	45–59	11,900 (26.0%)
	60–74	9875 (21.6%)
	75 and over	4350 (9.5%)
Level of exercise undertaken in past week		
	High	4920 (10.8%)
	Moderate	10,046 (22.0%)
	Low	14,514 (31.8%)
	Sedentary	16,218 (35.5%)
Alcohol exceeded drinking guidelines		
	No	33,867 (75.0%)
	Yes	11,284 (25.0%)
Frequency of alcohol consumption		
	Less than once per month	15,251 (33.8%)
	1 to 3 days per month	7983 (17.7%)
	1 to 2 days per week	9389 (20.8%)
	3 to 7 days per week	12,436 (27.6%)
Smoking status		
	Never smoked	22,619 (49.5%)
	Ex smoker	15,087 (33.0%)
	Current smoker	8011 (17.5%)
Income quintiles		
	First quintile (lowest 20%)	5754 (14.1%)
	Second quintile	9323 (22.8%)
	Third quintile	8552 (20.9%)
	Fourth quintile	8620 (21.1%)
	Fifth quintile (highest 20%)	8573 (21.0%)
Highest education level achieved		
	Year 11 and below	11,955 (26.2%)
	Year 12 or Certificate	16,621 (36.4%)
	Diploma or Advanced Diploma	4936 (10.8%)
	Bachelor or Postgraduate qualification	12,205 (26.7%)
Bodily pain experienced in the past four weeks		
	No pain	13,828 (30.2%)
	Very mild or mild pain	18,780 (41.1%)
	Moderate pain	8951 (19.6%)
	Severe or very severe pain	4158 (9.1%)
Number of Long-Term Current Conditions		
	No conditions	4465 (9.8%)
	1 to 2	14,874 (32.5%)
	3 to 5	15,635 (34.2%)
	6 or more	10,743 (23.5%)
Body Mass Index (BMI)		
	Underweight	527 (1.2%)
	Healthy weight	14,010 (32.5%)
	Overweight	15,541 (36.0%)
	Obese	13,080 (30.3%)
Self-assessed Health		
	Fair or poor	7586 (16.6%)
	Good	13,544 (29.6%)
	Very good	16,013 (35.0%)
	Excellent	8574 (18.8%)

**Table 2:** Bivariate relationships between population characteristics and psychological distress and sex—weighted *.

		Psychological Distress		χ^2^	*p*-Value	Sex		χ^2^	*p*-Value
	Item	Low or Moderate	High or Very High			Male	Female		
Psychological distress									
	Low distress					49.97	50.03	136.22	<0.001
	Moderate distress					41.60	58.40		
	High distress								
	Very high distress								
Usual daily servings of vegetables									
	1 or less	83.88	16.12	304.87	<0.001	56.40	43.60	474.55	<0.001
	2 servings	88.66	11.34			48.35	51.65		
	3 to 4 servings	90.05	9.95			44.72	55.28		
	5 or more	90.65	9.35			42.53	57.47		
Usual daily servings of fruit									
	1 or less	86.02	13.98	156.1	<0.001	53.88	46.12	480.72	<0.001
	2 servings	89.96	10.04			43.11	56.89		
	3 to 4 servings	89.71	10.29			44.34	55.66		
	5 or more	88.39	11.61			52.23	47.77		
Year									
	2012	89.13	10.87	52.88	<0.001	48.12	51.88	2.13	<0.001
	2015	88.19	11.81			49.14	50.86		
	2018	86.46	13.54			48.49	51.51		
Sex									
	Male	89.73	10.27	136.22	<0.001				
	Female	86.17	13.83						
Age									
	18–29	86.12	13.88	70.6	<0.001	50.86	49.14	39.05	<0.001
	30–44	88.58	11.42			48.81	51.19		
	45–59	87.16	12.84			48.69	51.31		
	60–74	89.06	10.94			49.03	50.97		
	75 and over	90.35	9.65			50.86	49.14		
Level of exercise undertaken in past week									
	High	93.32	6.68	612	<0.001	61.59	38.41	489.71	<0.001
	Moderate	91.53	8.47			51.13	48.87		
	Low	88.67	11.33			44.68	55.32		
	Sedentary	83.15	16.85			47.21	52.79		
Alcohol exceeded drinking guidelines									
	No	87.51	12.49	26.29	<0.001	42.78	57.22	2252.87	<0.001
	Yes	89.32	10.68			68.79	31.21		
Frequency of alcohol consumption									
	Less than once per month	84.57	15.43	286.16	<0.001	35.74	64.26	2157.43	<0.001
	1 to 3 days per month	87.88	12.12			46.96	53.04		
	1 to 2 days per week	90.34	9.66			55.55	44.45		
	3 to 7 days per week	90.44	9.56			62.90	37.10		
Smoking status									
	Never smoked	90.57	9.43	881.45	<0.001	42.10	57.90	925.2	<0.001
	Ex smoker	88.72	11.28			56.16	43.84		
	Current smoker	77.86	22.14			57.00	43.00		
Income quintiles									
	First quintile (lowest 20%)	82.61	17.39	972.73	<0.001	38.71	61.29	2462.72	<0.001
	Second quintile	81.29	18.71			38.51	61.49		
	Third quintile	87.32	12.68			43.92	56.08		
	Fourth quintile	91.41	8.59			55.53	44.47		
	Fifth quintile (highest 20%)	94.69	5.31			70.78	29.22		
Highest education level achieved									
	Year 11 and below	82.87	17.13	594.06	<0.001	43.18	56.82	613.95	<0.001
	Year 12 or Certificate	86.89	13.11			56.17	43.83		
	Diploma or Advanced Diploma	89.37	10.63			43.94	56.06		
	Bachelor or Postgraduate qualification	93.09	6.91			45.66	54.34		
Bodily pain experienced in the past four weeks									
	No pain	94.16	5.84	2597.38	<0.001	52.07	47.93	226.4	<0.001
	Very mild or mild pain	90.32	9.68			50.07	49.93		
	Moderate pain	81.38	18.62			44.79	55.21		
	Severe or very severe pain	66.42	33.58			40.66	59.34		
Number of long-term current conditions									
	No conditions	95.73	4.27	3037.22	<0.001	59.36	40.64	512.95	<0.001
	1 to 2	93.89	6.11			51.45	48.55		
	3 to 5	88.43	11.57			47.22	52.78		
	6 or more	72.10	27.90			41.30	58.70		
Body Mass Index (BMI) ^									
	Underweight	84.72	15.28	189.72	<0.001	32.87	67.13	1182.66	<0.001
	Healthy weight	88.97	11.03			39.37	60.63		
	Overweight	89.75	10.25			93.41	6.59		
	Obese	84.73	15.27			50.29	49.71		
Self-assessed Health ^									
	Fair or poor	64.12	35.88	4687.53	<0.001	49.26	50.74	10.38	0.086
	Good	87.46	12.54			50.00	50.00		
	Very good	93.39	6.61			48.29	51.71		
	Excellent	96.53	3.47			48.39	51.61		

* Results of chi-square tests are expressed as percentages of people with psychological distress within each item category, with chi-square and *p*-values provided. ^ Variables were not included in the final model.

**Table 3:** Vegetable and fruit consumption, lifestyle, and sociodemographic characteristics, and their association with psychological distress—multivariable logistic regression models with weighted population data.

		Model 1		Model 2		Model 3	
		OR (95%CI)	*p*	OR (95%CI)	*p*	OR (95%CI)	*p*
Usual daily serving of vegetables							
	1 or less	1.00 (Ref)		1.00 (Ref)		1.00 (Ref)	
	2 servings	0.68 (0.61–0.76)	<0.001	0.74 (0.65–0.83)	<0.001	0.77 (0.68–0.88)	<0.001
	3 to 4 servings	0.60 (0.54–0.67)	<0.001	0.66 (0.59–0.73)	<0.001	0.66 (0.58–0.75)	<0.001
	5 or more	0.56 (0.48–0.66)	<0.001	0.66 (0.56–0.78)	<0.001	0.61 (0.50–0.74)	<0.001
Usual daily serving of fruit							
	1 or less	1.00 (Ref)		1.00 (Ref)		1.00 (Ref)	
	2 servings	0.72 (0.65–0.80)	<0.001	0.82 (0.74–0.91)	<0.001	0.89 (0.80–0.99)	0.027
	3 to 4 servings	0.78 (0.68–0.88)	<0.001	0.90 (0.79–1.03)	0.11	0.92 (0.80–1.11)	0.22
	5 or more	0.91 (0.70–1.19)	0.47	0.96 (0.71–1.29)	0.78	1.03 (0.71–1.51)	0.86
Year							
	2012	1.00 (Ref)		1.00 (Ref)		1.00 (Ref)	
	2015	1.08 (0.98–1.20)	0.11	1.12 (1.02–1.24)	0.024	1.10 (0.99–1.22)	0.07
	2018	1.28 (1.18–1.39)	<0.001	1.33 (1.22–1.44)	<0.001	1.31 (1.19–1.44)	<0.001
Sex							
	Male	1.00 (Ref)		1.00 (Ref)		1.00 (Ref)	
	Female	1.53 (1.42–1.64)	<0.001	1.45 (1.34–1.58)	<0.001	1.08 (0.98–1.19)	0.13
Age							
	18–29	1.00 (Ref)		1.00 (Ref)		1.00 (Ref)	
	30–44	0.82 (0.72–0.94)	0.006	0.75 (0.65–0.87)	<0.001	0.75 (0.65–0.88)	<0.001
	45–59	0.98 (0.86–1.13)	0.79	0.85 (0.74–0.98)	0.030	0.43 (0.36–0.50)	<0.001
	60–74	0.85 (0.74–0.98)	0.027	0.75 (0.64–0.88)	<0.001	0.19 (0.17–0.23)	<0.001
	75 and over	0.73 (0.61–0.88)	0.001	0.58 (0.48–0.71)	<0.001	0.11 (0.09–0.14)	<0.001
Level of exercise undertaken in past week							
	High			1.00 (Ref)		1.00 (Ref)	
	Moderate			1.25 (1.05–1.49)	0.012	1.16 (0.95–1.42)	0.14
	Low			1.60 (1.35–1.89)	<0.001	1.31 (1.08–1.59)	0.007
	Sedentary			2.24 (1.89–2.65)	<0.001	1.61 (1.33–1.96)	<0.001
Frequency of alcohol consumption							
	Less than once per month			1.00 (Ref)		1.00 (Ref)	
	1 to 3 days per month			0.78 (0.70–0.86)	<0.001	0.85 (0.76–0.96)	0.01
	1 to 2 days per week			0.62 (0.55–0.70)	<0.001	0.77 (0.67–0.88)	<0.001
	3 to 7 days per week			0.60 (0.54–0.66)	<0.001	0.74 (0.66–0.83)	<0.001
Smoking status							
	Never smoked			1.00 (Ref)		1.00 (Ref)	
	Ex-smoker			1.48 (1.34–1.64)	<0.001	1.20 (1.08–1.35)	0.002
	Current smoker			2.75 (2.48–3.04)	<0.001	2.07 (1.82–2.36)	<0.001
Income quintiles							
	First quintile (lowest 20%)					1.00 (Ref)	
	Second quintile					0.99 (0.87–1.12)	0.84
	Third quintile					0.67 (0.59–0.77)	<0.001
	Fourth quintile					0.49 (0.42–0.57)	<0.001
	Fifth quintile (highest 20%)					0.39 (0.33–0.47)	<0.001
Highest education level achieved							
	Year 11 and below					1.00 (Ref)	
	Year 12 or Certificate					0.81 (0.73–0.91)	<0.001
	Diploma or Advanced Diploma					0.79 (0.65–0.94)	0.011
	Bachelor’s or Postgraduate qualification					0.63 (0.53–0.73)	<0.001
Bodily pain experienced in the past four weeks							
	No pain					1.00 (Ref)	
	Very mild or mild pain					1.40 (1.21–1.61)	<0.001
	Moderate pain					2.23 (1.89–2.62)	<0.001
	Severe or very severe pain					4.01 (3.42–4.69)	<0.001
Number of Long-Term Current Conditions							
	No conditions					1.00 (Ref)	
	1 to 2					1.93 (1.49–2.49)	<0.001
	3 to 5					4.30 (3.37–5.47)	<0.001
	6 or more					12.33 (9.42–16.13)	<0.001

Model 1: adjusted for year, sex, and age; Model 2: adjusted for year, sex, age, exercise level, frequency of alcohol consumption, and smoking status; Model 3: adjusted for year, sex, age, exercise level, frequency of alcohol consumption, smoking status, income, education, bodily pain, and number of current long-term conditions. OR = odds ratio; CI = confidence interval.

**Table 4:** Multivariable logistic regression models with weighted population data by sex.

		Male						Female					
		Model 1		Model 2		Model 3		Model 1		Model 2		Model 3	
		OR (95%CI)	*p*	OR (95%CI)	*p*	OR (95%CI)	*p*	OR (95%CI)	*p*	OR (95%CI)	*p*	OR (95%CI)	*p*
Usual daily serving of vegetables													
	1 or less		1.00 (Ref)	1.00 (Ref)		1.00 (Ref)		1.00 (Ref)		1.00 (Ref)		1.00 (Ref)	
	2 serving	0.66 (0.56–0.77)	<0.001	0.79 (0.60–0.84)	<0.001	0.81 (0.66–0.98)	0.033	0.68 (0.59–0.78)	<0.001	0.74 (0.64–0.85)	<0.001	0.73 (0.62–0.86)	0.66 (0.56–0.77)
	3 to 4 serving	0.67 (0.56–0.791)	<0.001	0.72 (0.60–0.86)	<0.001	0.74 (0.59–0.94)	0.014	0.55 (0.49–0.63)	<0.001	0.61 (0.54–0.70)	<0.001	0.60 (0.52–0.68)	0.67 (0.56–0.791)
	5 or more	0.76 (0.57–1.00)	0.05	0.86 (0.64–1.15)	0.29	0.87 (0.61–1.25)	0.45	0.46 (0.37–0.56)	<0.001	0.55 (0.44–0.68)	<0.001	0.47 (0.38–0.59)	0.76 (0.57–1.00)
Usual daily serving of fruit													
	1 or less	1.00 (Ref)		1.00 (Ref)		1.00 (Ref)		1.00 (Ref)		1.00 (Ref)		1.00 (Ref)	
	2 servings	0.80 (0.68–0.93)	0.005	0.90 (0.76–1.06)	0.20	0.99 (0.84–1.18)	0.92	0.67 (0.59, 0.765)	<0.001	0.78 (0.68–0.89)	<0.001	0.83 (0.71–0.97)	0.017
	3 to 4 servings	0.80 (0.64–1.00)	0.046	0.93 (0.74–1.16)	0.49	0.94 (0.73, 1.22)	0.64	0.76 (0.67–0.86)	<0.001	0.88 (0.77–1.00)	0.05	0.90 (0.76–01.06)	0.21
	5 or more	1.10 (0.69–1.75)	0.68	1.15 (0.71–1.86)	0.58	1.28 (0.70–2.34)	0.41	0.75 (0.55–1.01)	<0.001	0.79 (0.55–1.13)	0.19	0.83 (0.55–1.253)	0.37
Year													
	2012	1.00 (Ref)		1.00 (Ref)		1.00 (Ref)		REF		1.00 (Ref)		REF	
	2015	1.10 (0.95–1.28)	0.18	1.13 (0.97–1.31)	0.11	1.15 (0.97–1.35)	0.10	1.07 (0.94–1.22)	0.28	1.13 (0.99–1.28)	0.07	1.09 (0.95–1.24)	0.21
	2018	1.39 (1.20–1.60)	<0.001	1.41 (1.22–1.63)	<0.001	1.39 (1.17– 1.66)	<0.001	1.22 (1.09–1.35)	0.001	1.28 (1.14–1.43)	<0.001	1.28 (1.10–1.48)	0.001
Age													
	18–29	1.00 (Ref)		1.00 (Ref)		1.00 (Ref)		1.00 (Ref)		1.00 (Ref)		1.00 (Ref)	
	30–44	0.88 (0.79–1.28)	0.27	0.79 (0.63–1.00)	0.05	0.88 (0.68–1.14)	0.34	0.79 (0.67–0.92)	0.004	0.73 (0.62–0.86)	<0.001	0.69 (0.57–0.82)	<0.001
	45–59	0.98 (0.79–1.22)	0.84	0.84 (0.67–1.05)	0.13	0.44 (0.34–0.58)	<0.001	0.99 (0.85–1.17)	0.94	0.87 (0.74–1.03)	0.10	0.42 (0.34–0.50)	<0.001
	60–74	0.89 (0.73–1.10)	0.28	0.76 (0.60–0.95)	0.019	0.19 (0.15–0.24)	<0.001	0.83 (0.70–0.98)	0.032	0.75 (0.63–0.90)	0.002	0.20 (0.16–0.24)	<0.001
	75 and over	0.78 (0.59–1.03)	0.08	0.60 (0.44–0.82)	0.002	0.12 (0.08–0.17)	<0.001	0.69 (0.54–0.90)	0.006	0.56 (0.42–0.73)	<0.001	0.10 (0.08–0.14)	<0.001
Level of exercise undertaken in past week													
	High			1.00 (Ref)		1.00 (Ref)				1.00 (Ref)		1.00 (Ref)	
	Moderate			1.19 (0.94–1.51)	0.14	1.10 (0.86–1.41)	0.043			1.36 (1.05–1.75)	0.02	1.27 (0.97–1.67)	0.09
	Low			1.58 (1.27–1.95)	<0.001	1.32 (1.02–1.7)	0.036			1.68 (0.64–0.91)	0.001	1.37 (1.01–1.87)	0.045
	Sedentary			2.04 (1.65–2.52)	<0.001	1.42 (1.12–1.82)	0.005			2.49 (1.91–3.24)	<0.001	1.86 (1.39–2.48)	<0.001
Frequency of alcohol consumption													
	Less than once per month			1.00 (Ref)		1.00 (Ref)				1.00 (Ref)		1.00 (Ref)	
	1 to 3 days per month			0.66 (0.55–0.78)	<0.001	0.73 (0.59–0.91)	0.005			0.86 (0.75–0.97)	0.019	0.94 (0.81–1.08)	0.35
	1 to 2 days per week			0.47 (0.40–0.54)	<0.001	0.59 (0.50, 0.71)	<0.001			0.76 (0.64–0.91)	0.003	0.94 (0.77–1.14)	0.50
	3 to 7 days per week			0.50 (0.43–0.57)	<0.001	0.62 (0.53–0.72)	<0.001			0.69 (0.60–0.80)	<0.001	0.86 (0.73–1.01)	0.07
Smoking status													
	Never smoked			1.00 (Ref)		1.00 (Ref)				1.00 (Ref)		1.00 (Ref)	
	Ex-smoker			1.57 (1.33–1.86)	<0.001	1.22 (0.99–1.50)	0.062			1.39 (1.24–1.56)	<0.001	1.15 (1.0–1.30)	0.035
	Current smoker			2.78 (2.35–3.17)	<0.001	2.01 (1.66–2.44)	<0.001			2.77 (2.43–3.15)	<0.001	1.10 (1.80–2.46)	<0.001
Income quintiles													
	First quintile (lowest 20%)					1.00 (Ref)						1.00 (Ref)	
	Second quintile					1.05 (0.85–1.29)	0.66					0.95 (0.81–1.12)	0.54
	Third quintile					0.66 (0.53–0.84)	0.001					0.68 (0.56–0.81)	<0.001
	Fourth quintile					0.43 (0.33–0.57)	<0.001					0.55 (0.45–0.66)	<0.001
	Fifth quintile (highest 20%)					0.36 (0.27–0.47)	<0.001					0.44 (0.35–0.56)	<0.001
Highest education level achieved													
	Year 11 and below					1.00 (Ref)						1.00 (Ref)	
	Year 12 or Certificate					0.83 (0.71–0.96)	<0.001					0.80 (0.68–0.93)	0.005
	Diploma or Advanced Diploma					0.87 (0.65–1.16)	<0.001					0.73 (0.59–0.89)	0.003
	Bachelor or Postgraduate qualification					0.71 (0.57–0.88)	<0.001					0.57 (0.47–0.70)	<0.001
Bodily pain experienced in the past four weeks													
	No pain					1.00 (Ref)						1.00 (Ref)	
	Very mild or mild pain					1.49 (1.21–1.83)	<0.001					1.35 (1.11–1.64)	0.003
	Moderate pain					2.43 (1.93–3.06)	<0.001					2.10 (1.73–2.55)	<0.001
	Severe or very severe pain					4.91 (3.83–6.29)	<0.001					3.51 (2.90–4.24)	<0.001
Number of Long-Term Current Conditions													
	No conditions					1.00 (Ref)						1.00 (Ref)	
	1 to 2					1.82 (1.28–2.60)	0.001					2.03 (1.35–3.05)	0.001
	3 to 5					3.98 (2.76–5.73)	<0.0001					4.61 (3.21–6.61)	<0.001
	6 or more					12.05 (8.18–17.73)	<0.001					12.70 (8.48–19.02)	<0.001

Model 1: adjusted for year, sex, and age; Model 2: adjusted for year, sex, age, exercise level, frequency of alcohol consumption, and smoking status; Model 3: adjusted for year, sex, age, exercise level, frequency of alcohol consumption, smoking status, income, education, bodily pain, and number of current long-term conditions. OR = odds ratio; CI = confidence interval.

## Data Availability

Restrictions apply to the availability of these data. Data were obtained from the Australian Bureau of Statistics and are available at: https://microdatadownload.abs.gov.au/MicrodataDownload/login.xhtml (accessed 24 July 2024).

## Supplementary Materials

The following supporting information can be downloaded at https://www.mdpi.com/article/10.3390/ijerph22071037/s1, Table S1: Select population characteristics by fruit and vegetable consumption guideline adherence.

## Abbreviations

The following abbreviations are used in this manuscript:

ABS	Australian Bureau of Statistics.
BMI	Body Mass Index.
K10	Kessler Psychological Distress Scale.
NHMRC	National Health and Medical Research Council.
NHS	National Health Survey.
SES	Socioeconomic Status.
UPF	Ultra-Processed Foods
