# Supplementary material for: Vegetable and Fruit Consumption and Psychological Distress: Findings from Australian National Health Survey Data, 2011–2018
Source: Int J Environ Res Public Health. 2025 Jun 28;22(7):1037. doi: 10.3390/ijerph22071037 (PMC12294948; doi:10.3390/ijerph22071037)
Supplement: Supplementary file 1 [file ijerph-22-01037-s001.zip › ijerph-3588638-supplementary.pdf]

Table S1. Select population characteristics by fruit and vegetable consumption guideline adherence.

	Met fruit and vegetable guidelines	Met vegetable guidelines only	Met fruit guidelines only	Did not meet fruit or vegetable guidelines
**Year**				
2012	4.30	1.95	43.87	49.88
2015	5.12	1.93	44.67	48.27
2018	5.43	2.23	45.87	46.48
**Psychological Distress**				
Low/moderate distress	5.19	2.07	45.67	47.08
High/Very high distress	3.31	1.82	38.63	56.24
**Sex**				
Male	2.57	1.04	42.17	54.22
Female	7.25	2.98	47.37	42.40
**Age**				
18-29	3.15	1.48	39.77	55.60
30-44	3.97	2.13	40.74	53.16
45-59	5.01	2.23	45.37	47.40
60-74	7.34	2.26	52.02	38.38
75+	7.92	2.01	55.30	34.77
**Highest Educational Attainment**				
Year 11 and below	5.03	1.89	44.32	48.75
Year 12 or Certificate	4.16	1.81	42.64	51.38
Diploma or Advanced Diploma	5.69	2.66	44.68	46.97
Bachelor or Postgraduate qualification	5.60	2.25	48.45	43.70
**Income Quintile**				

First quintile (lowest 20%)	5.42	1.87	45.45	47.26
Second quintile	6.19	2.45	46.81	44.56
Third quintile	4.83	2.08	43.26	49.83
Fourth quintile	4.13	1.80	43.09	50.97
Fifth quintile (highest 20%)	3.49	1.97	44.78	49.75
**Smoking Status**				
Never smoked	5.53	1.84	48.65	43.98
Ex smoker	5.14	2.33	45.74	46.80
Current smoker	2.80	2.13	30.84	64.23
**Alcohol Consumption Exceeded Guidelines**				
No	5.35	1.96	47.11	45.59
Yes	3.82	2.35	38.51	55.31
**Frequency of Alcohol Consumption**				
Less than once per month	5.66	1.80	48.22	44.33
1 to 3 days per month	5.06	1.94	44.94	48.06
1 to 2 days per week	4.39	1.99	44.48	49.15
3 to 7 days per week	4.45	2.51	40.39	52.65
**Level of Exercise Undertaken in Past Week**				
High	6.77	2.03	50.89	40.30
Moderate	6.63	2.16	49.22	41.99
Low	4.54	2.06	45.52	47.87
Sedentary	3.66	1.92	39.43	54.99
**Body Mass Index**				
Underweight	3.70	2.09	36.99	57.22

Healthy weight	5.56	2.13	44.81	47.50
Overweight	4.86	1.81	46.17	47.16
Obese	4.67	2.22	43.15	49.97
**Bodily Pain in the Last Four Weeks**				
No pain	4.95	1.82	44.96	48.28
Very mild or mild pain	4.77	1.96	45.18	48.09
Moderate pain	5.02	2.48	44.14	48.35
Severe or very severe pain	5.86	2.32	43.96	47.86
**Number of Long-Term Current Conditions**				
No conditions	4.10	1.17	44.40	50.33
1 to 2	4.26	1.79	43.99	49.96
3 to 5	5.25	2.42	45.11	47.22
6 or more	10.57	4.00	77.77	7.66
**Self-Assessed Health**				
Fair or Poor	4.20	2.10	37.83	55.87
Good	4.10	1.82	41.12	52.96
Very good	4.99	2.09	46.58	46.33
Excellent	6.99	2.35	49.31	41.35
